# CDX2 and Reg IV expression and correlation in gastric cancer

**DOI:** 10.1186/s12876-021-01678-9

**Published:** 2021-02-27

**Authors:** Dandan Chai, Huifen Du, Kesheng Li, Xueliang Zhang, Xiaoqin Li, Xiaoning Zhao, Xiaowen Lian, Yang Xu

**Affiliations:** 1 Department of Medicine Biotechnology, Gansu Provincial Academic Institute for Medical Research, Xiaoxihu East Street No. 2, Lanzhou, 730050 Gansu China; 2 Department of Internal Medicine, Gansu Provincial Cancer Hospital, Lanzhou, Gansu China; 3 Department of Pathology, Gansu Provincial Cancer Hospital, Lanzhou, Gansu China; 4 Department of Surgery, Gansu Provincial Cancer Hospital, Lanzhou, Gansu China

**Keywords:** CDX2, Reg IV, Correlation, Migration, Invasion, Gastric cancer

## Abstract

**Background:**

Ectopic expression of CDX2 is associated with the development and progression of gastric cancer. Previous studies showed that CDX2 may be an upstream regulator of Reg IV expression in gastric cancer, and our previous report showed that Reg IV upregulated SOX9 expression and enhanced cell migration and invasion in gastric cancer cells. However, the regulatory roles of CDX2 have not been clarified in gastric cancer, and the correlation between CDX2 and Reg IV requires further study.

**Methods:**

CDX2 and Reg IV were examined in gastric cancer specimens and paired adjacent tissues via real-time PCR and immunohistochemistry (IHC). The association between CDX2 and Reg IV was assessed using the χ^2^-test and Spearman’s rank correlation. To verify their relationship, knockdown and exogenous expression of CDX2 or Reg IV were performed in AGS and MKN-45 gastric cancer cells, and their expression was subsequently analyzed via a real-time PCR and western blotting. Wound-healing and Transwell assays were used to examine migration and invasion in AGS and MKN-45 cells following CDX2 silencing or overexpression.

**Results:**

A positive correlation was observed between CDX2 and Reg IV expression at the mRNA and protein levels in gastric cancer tissues. CDX2 silencing significantly downregulated Reg IV expression, and CDX2 overexpression significantly upregulated Reg IV expression in AGS and MKN-45 cells. Neither Reg IV silencing nor overexpression had any effect on CDX2 protein expression in AGS or MKN-45 cells, even though both affected the expression of CDX2 mRNA. Functionally, CDX2 silencing significantly inhibited cell migration and invasion, and CDX2 overexpression significantly promoted cell migration and invasion in AGS and MKN-45 cells.

**Conclusions:**

Our findings demonstrate that CDX2 expression was positively correlated with that of Reg IV in gastric cancer, and CDX2 promoted cell migration and invasion through upregulation of Reg IV expression in AGS and MKN-45 cells.

## Background

Gastric cancer is one of the most common cancers and the leading cause of cancer-related deaths worldwide. Caudal type homeobox 2 (CDX2) is an intestine-specific transcription factor belonging to the caudal-related homeobox gene family. Although CDX2 is not expressed in the gastric mucosa under normal conditions, its ectopic expression has been detected in gastric intestinal metaplasia (IM) and gastric carcinomas [1–8]. Reports have shown that CDX2 expression is related to IM of the stomach, lymphatic metastasis, survival, cell proliferation, migration, invasion, and multidrug resistance (MDR) in gastric carcinoma [1, 2, 6, 8–31]. Several studies showed that CDX2 expression may promote or contribute to IM in gastric cells [1, 2, 9–11]. The same results were observed in CDX2 transgenic mouse models [12–14] and long-term IM-induced invasive gastric carcinoma [15]. Moreover, increased CDX2 expression was associated with lymph node metastasis in gastric adenocarcinoma [16] and a more invasive phenotype in AGS cells [17]. In addition, CDX2 overexpression promoted the development of MDR in SGC-7901/DDP (cisplatin-resistant) and BGC-823/5-FU (5-fluorouracil-resistant) human gastric cancer cells in vitro and in vivo [18]. Alternatively, CDX2 silencing significantly inhibited the growth of MGC-803 human gastric cancer cells [19] and reversed the progression of MDR in SGC-7901/DDP cells in vitro and in vivo [20]. These studies suggest that CDX2 may possess oncogenic activity. Conversely, several studies have indicated that CDX2 may act as a tumor suppressor. Reports have shown that CDX2 expression is negatively correlated with lymph node metastasis, tumor invasion, and survival in gastric cancer [6–8, 21–25]. Additionally, CDX2 overexpression inhibited proliferation, migration, and invasion in human gastric cancer cell lines (MGC-803, MKN-45, and BGC-823) [26–31]. Alternatively, CDX2 silencing promoted migration and invasion in NCI-N87 cells [26]. However, Dang et al. concluded that disruption of CDX2 did not significantly affect tumorigenic potential [32]. Taken together, these conflicting results show that the regulatory roles of CDX2 in gastric cancer remain unclear.

*Regenerating islet-derived family member 4* (*REG4*) encodes the Reg IV protein and is a member of the *REG* gene family, which belongs to the calcium-dependent lectin superfamily. Reg IV is a small secretory protein that is highly expressed in gastrointestinal malignancies, indicating that Reg IV may have a prognostic or predictive value in these cancers [33–35]. Moreover, several studies revealed that Reg IV expression is related to metastasis, migration, proliferation, apoptosis, and drug resistance in gastric cancer [36–40]. Wang et al. established two stably transfected cell lines (SGC-7901/Reg IV and MKN-45/Reg IV) and suggested that Reg IV interacts with G protein-coupled receptor 37 (GPR37) in gastric cancer cells to promote peritoneal metastasis by increasing cell adhesion [36]. Huang et al. reported that Reg IV promoted the growth, proliferation, and migration of MKN-45 cells through the Akt pathway [37]. Recent studies showed that Reg IV inhibited apoptosis through activation of Erk/Bim signaling, thereby enhancing the resistance of AGS and SGC-7901 cells to 5-FU [38]. Another study showed that the single-chain antibody against Reg IV significantly inhibited cell proliferation and enhanced 5-FU-induced cell death in MKN-45 and AGS cells [39]. These findings indicate that Reg IV may be a tumor-promoting factor in gastric cancer. Our previous study suggested that Reg IV upregulated SRY related high-mobility group box 9 (SOX9) expression and enhanced cell migration and invasion in MKN-45 and AGS cells [40]. Additionally, CDX2 may be an upstream regulator of Reg IV expression in gastric cancer [41, 42]. Therefore, we speculate that CDX2 may regulate the migration and invasion of gastric cancer cells through Reg IV/SOX9 signaling. In this study, we investigated the correlation between CDX2 and Reg IV in gastric cancer tissues and cell lines, and the effect of CDX2 on migration and invasion in gastric cancer cells in vitro.

## Methods

### Tissue samples

Formalin-fixed paraffin-embedded tissue specimens from 102 primary gastric cancer patients who received surgery in the Gansu Provincial Cancer Hospital from March 2014 to April 2015 were subjected to immunohistochemical analysis. Another 93 cases and paired adjacent tissues were obtained from surgical specimens resected from patients who had not received chemotherapy or radiotherapy prior to surgery. The samples were immediately frozen in liquid nitrogen and stored at − 80 °C for preparation of total RNA. Detailed clinicopathologic characteristics of the patients were listed in Table 1. All of the patients gave written informed consent, and the procedures involving human tissues were approved by the Medical Ethics Committee of Gansu Provincial Academic Institute for Medical Research.

**Table 1:** Clinicopathologic characteristics of the patients

Clinicopathologic parameter		IHC staining	Real-time PCR
All cases		102	93
Age (years)	< 60	61	48
	≥ 60	41	45
Gender	Male	76	70
	Female	26	23
Depth of invasion	T1 + T2	27	22
	T3 + T4	75	71
Lymph node metastasis	Negative	32	22
	Positive	70	71
TNM staging	I + II	43	29
	III + IV	59	64

### Immunohistochemistry

Formalin-fixed, paraffin-embedded tissue specimens were obtained and handled according to standard procedures. Serial 4-μm sections were prepared and stained using biotin-streptavidin HRP detection systems (ZSGB-BIO, Beijing, China) with the following primary antibodies: CDX2 (1:300; Bioss, Beijing, China), Reg IV (1:300; Bioss). Immunoreactivity was visualized with 3,3′-diaminobenzidine (DAB). Nuclei were lightly counterstained with hematoxylin. Staining intensity was scored using the following scale: negative, 0; weak, 1; moderate, 2; and strong, 3. The proportion of stained cells was scored using the following scale: < 5%, 0; 5%–25%, 1; 25%–50%, 2; 50%–75%, 3; and > 75%, 4. The scores for staining intensity and proportion of positive cells were multiplied. For the purpose of statistical analysis, tumors with a final staining score ≥ 3 were considered to be positive. Images were acquired using RPT-PathQC system software (Logene, Wuxi, China) under an Olympus BX43 microscope (200× magnification).

### Total RNA extraction and real-time PCR

Total RNA was extracted using RNAiso Plus (Takara, Dalian, China) and reverse-transcribed using PrimeScript RT Master Mix (Takara) according to the manufacturer’s protocol. We amplified cDNA samples using SYBR Premix Ex Taq II (Tli RNaseH Plus) (Takara) and determined the cycle threshold (Ct). Sequences of the primers used are shown in Table 2. Relative mRNA expression levels were calculated using the 2^−ΔΔCt^ method and the Ct values were normalized using GAPDH as the internal control.

**Table 2:** Real-time PCR primers

Primers	Forward	Reverse
CDX2	5′-AAGTGAAAACCAGGACGAAAGA-3'	5′-GGATGGTGATGTAGCGACTGTA-3'
Reg IV	5′-CAGATCCTGGTCTGGCAAGT-3'	5′-ATTCGTTGCTGCTCCAAGTT-3'
GAPDH	5′-GCACCGTCAAGGCTGAGAAC-3'	5′-TGGTGAAGACGCCAGTGGA-3'

### Cell culture

The human gastric cancer AGS cells were purchased from the Cell Bank of the Chinese Academy of Sciences (Shanghai, China), and MKN-45 cells were kindly provided by Prof. Hongyun Guo from Gansu Provincial Academic Institute for Medical Research. Cells were cultured in high-glucose DMEM (Thermo Fisher Scientific, Shanghai, China) with 10% fetal bovine serum (FBS, Minhai Biotechnology Co., Ltd., Beijing, China) at 37 °C with 5% CO_2_.

### Silencing of *CDX2* or *Reg IV* genes by siRNAs

Chemically synthetic siRNAs for human CDX2, Reg IV, and a nontargeting control siRNA were purchased from GenePharma Co., Ltd. (Shanghai, China). Briefly, cells grown on six-well plates were transfected using 10–40 nM siRNA and 7.5 μL Lipofectamine RNAiMAX (Thermo Fisher Scientific) per well, and the medium was changed after 6 h. The knockdown efficiency was evaluated 48 h later via real-time PCR and western blotting. The sense and antisense strands of siRNAs are listed in Table 3.

**Table 3:** siRNA sequences

Name	Sequence
siR-CDX2-1	Sense, 5′-GACAAGGACGUGAGCAUGUTT-3'
	Antisense, 5′-ACAUGCUCACGUCCUUGUCTT-3'
siR-CDX2-2	Sense, 5′-GACAAAUAUCGAGUGGUGUTT-3'
	Antisense, 5′-ACACCACUCGAUAUUUGUCTT-3'
siR-CDX2-3	Sense, 5′-CAUCACCAUCCGGAGGAAATT-3'
	Antisense, 5′-UUUCCUCCGGAUGGUGAUGTT-3'
siR-Reg IV	Sense, 5′-CUUCAGGAAGCUGAGGAACTT-3'
	Antisense, 5′-GUUCCUCAGCUUCCUGAAGTT-3'
siR-NC	Sense, 5′-UUCUCCGAACGUGUCACGUTT-3'
	Antisense, 5′-ACGUGACACGUUCGGAGAATT-3'

## Overexpression of *CDX2* and *Reg IV* genes

The mammalian pEGFP-C1-Reg IV expression plasmid was described previously [40]. The CDX2 expression plasmid was constructed by inserting the coding sequence region of the human CDX2 cDNA into the pcDNA4/TO vector using the *Hin*d III and *Bam*H I endonucleases (TaKaRa) and verified by DNA sequencing (GENEWIZ, Suzhou, China). The CDX2 primer sequences were as follows: forward, 5′-CCCAAGCTTGCCACCATGTACGTGAGCTAC-3′; reverse, 5′-CGGGATCCTCACTGGGTGACGGTGGG-3′. The recombinant vector was designated pcDNA4/TO-CDX2, which was subsequently extracted with an E.Z.N.A. Endo-Free Plasmid Mini Kit (Omega Bio-Tek, Norcross, GA, USA). For plasmid transfection experiments, cells were transiently transfected using Lipofectamine 3000 (Thermo Fisher Scientific), as recommended by the manufacturer. Briefly, cells were seeded in six-well plates at a density of 4.25 × 10^5^ per well. After 24 h, cells were transfected using 2.5 μg plasmids and 7.5 μL Lipofectamine 3000 per well and medium was changed after 6 h. The empty vectors were used as the control. At 36 h post-transfection, the cells were lysed and analyzed using real-time PCR and western blotting.

### Western blotting

Total proteins were extracted using the Tissue or Cell Total Protein Extraction Kit (Sangon Biotech, Shanghai, China) and quantified with the BCA Protein Assay Kit (Sangon Biotech). Proteins (60–100 µg per lane) were separated via 12% SDS–PAGE and transferred to a PVDF membrane. After blocking with TBST containing 5% nonfat milk powder, the membranes were incubated with primary antibodies against CDX2 (1:1000; Abcam, Shanghai, China), Reg IV (1:1000; Cell Signaling Technology, Shanghai, China), and β-actin (1:1000; Santa Cruz Biotechnology, Shanghai, China) at 4 °C overnight. After washing with TBST, the membranes were incubated with the corresponding HRP-conjugated secondary antibody (Santa Cruz Biotechnology) at room temperature for 90 min. Immunoreactive bands were visualized with SuperSignal West Pico chemiluminescent substrate (Thermo Fisher Scientific) and recorded with a ChemiDoc™ XRS + (Bio-Rad). Signal intensities were subsequently quantified using Image Lab quantification software (Bio-Rad, Hercules, CA, USA).

### Wound-healing assay

Cells were seeded in six-well plates, and transfected with siRNAs, pcDNA4/TO-CDX2 or pEGFP-C1-Reg IV. When the cells reached 90% confluence, monolayers were scratched with a 200 μL pipette tip to create wounds and then rinsed twice with DMEM to remove suspended cells. Fresh DMEM with 1% FBS was added to the wounded cell layer, and the wound healing process was monitored at the indicated times (0, 24 and 36 h for AGS cells; 0, 24, 48 and 72 h for MKN-45 cells) with an inverted light microscope (Olympus CKX41) at 100 × magnification. Image J software was used to calculate the scratch area.

### Transwell assay

Transwell invasion and migration assays were performed using 24-well Falcon Cell Culture Inserts (8 μm PET track-etched membrane, Corning, USA). Cells (1 × 10^5^) were seeded into the upper chamber in serum-free DMEM. For invasion assays, Matrigel Matrix (Corning, USA) was diluted in serum-free DMEM to a final concentration of 100–150 μg/mL. The insert was then coated with the Matrigel and incubated at 37 °C overnight prior to the invasion assay. DMEM (500 μL) with 20% FBS was added to the lower chamber and cells were further incubated at 37 °C in 5% CO_2_ for 24 h (AGS) or 48 h (MKN-45). The invasive cells on the lower surface of the membrane were fixed and stained with a crystal violet solution. Cells were counted in five random fields/well under a light microscope (Olympus CKX41) at 200× magnification. Migration assay was performed under the same conditions as the invasion assay described above, except that the insert was not coated with Matrigel and DMEM with 10% FBS was added to the lower chamber.

### Statistical analysis

The association between CDX2 and Reg IV in gastric cancer tissues was analyzed using χ^2^-test and Spearman’s rank correlation. Data from cell-based studies are presented as mean ± SD of three independent experiments and statistically analyzed by a two-tailed Student’s *t*-test. All of the statistical analyses were carried out using SPSS 23.0, and *p* < 0.05 (*) or *p* < 0.01 (**) were considered to be statistically significant.

## Results

### CDX2 expression was positively correlated with Reg IV expression in gastric cancer tissues

**Fig. 1:**
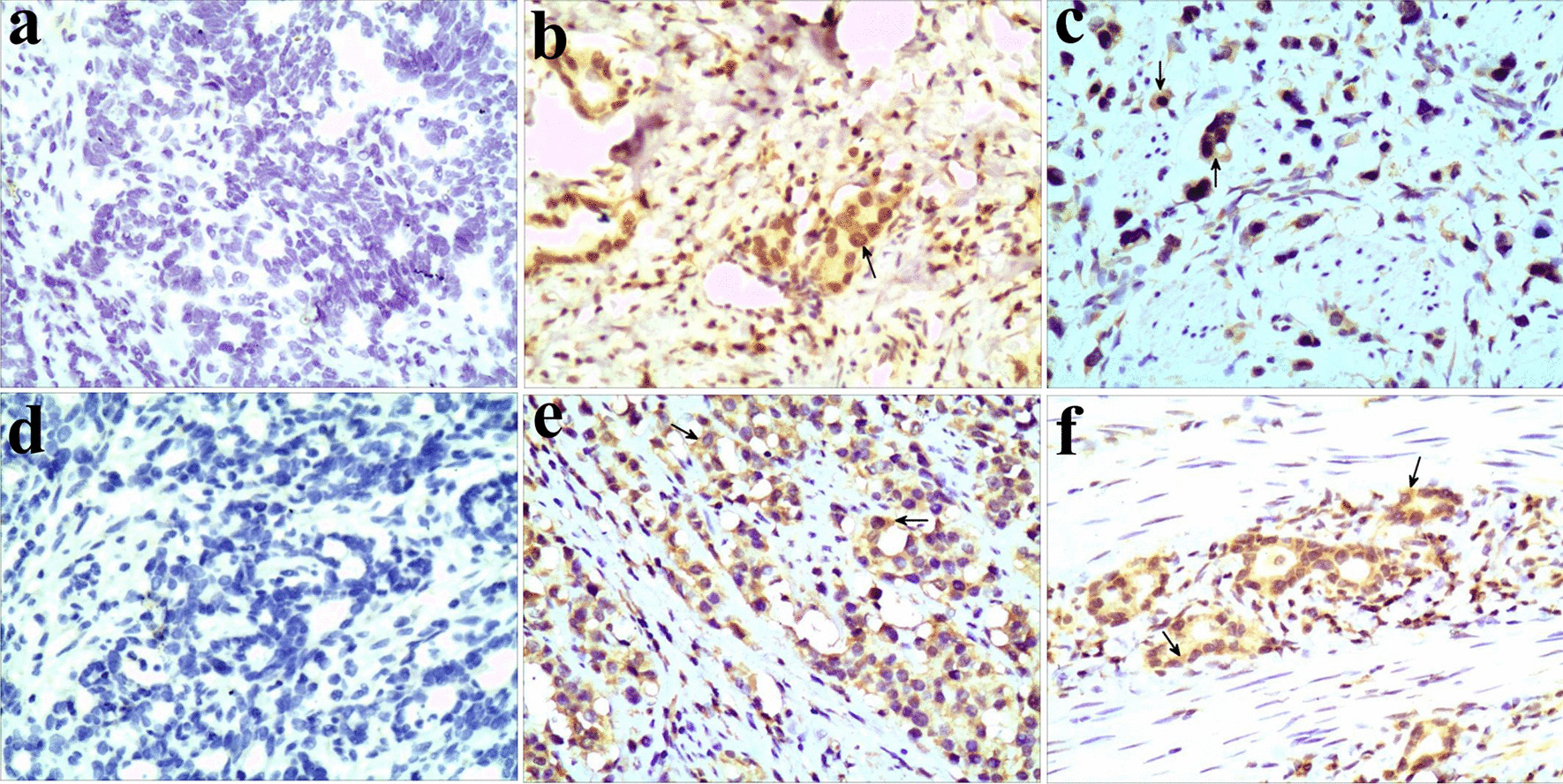
Representative images of immunohistochemical staining for CDX2 and Reg IV in gastric cancer tissues. **a** Negative staining of CDX2. **b** Nuclear staining of CDX2. **c** Nuclear and cytoplasmic staining of CDX2. **d** Negative staining of Reg IV. **e**, **f** Cytoplasmic staining of Reg IV. Magnification 200 ×

To explore the possible relationship between CDX2 and Reg IV in gastric cancer tissues, mRNA and protein levels were measured by real-time PCR and IHC. The samples were divided into two groups based on the relative expression of mRNA in the 93 cases: low (≤ 2) and high expression (> 2). A high expression of CDX2 mRNA was found in 31 of the 93 cases, and a low expression was found in 62 of the 93 cases. Low CDX2 expression was frequently observed in gastric cancer tissues with low Reg IV expression (72.6%, 45 of 62 cases), and high CDX2 expression was frequently observed in tissues with high Reg IV expression (67.7%, 21 of 31 cases; *p* = 0.0002 by χ^2^-test; Table 4). Correlation analysis further showed that the expression of CDX2 mRNA was positively associated with that of Reg IV mRNA in gastric cancer tissues (r = 0.387, *p* = 0.0001; Table 4). Furthermore, we examined protein expression in the 102 cases, and representative images of the IHC results are shown in Fig. 1. CDX2 was mainly found in the nucleus with cytoplasmic staining occasionally observed. Reg IV was detected mainly in the cytoplasm of tumor cells. CDX2 was negative in 67 cases and positive in 35 cases, and CDX2 expression was associated with Reg IV expression (*p* = 0.032 by χ^2^-test; Table 5). Further analysis showed that the expression of the CDX2 protein was positively associated with that of the Reg IV protein in gastric cancer tissues (r = 0.334, *p* = 0.001; Table 5). These results suggest that CDX2 expression positively correlates with Reg IV expression in gastric cancer tissues.

**Table 4:** Correlation between CDX2 mRNA and Reg IV mRNA in gastric cancer tissues

		Reg IV		Total	χ^2^	*p*	r	*p*
		Low	High					
CDX2	Low	45	17	62	13.906	0.0002**	0.387	0.0001**
		(72.6%)	(27.4%)					
	High	10	21	31				
		(32.3%)	(67.7%)

**Table 5:** Correlation between CDX2 protein and Reg IV protein in gastric cancer tissues

		Reg IV		Total	χ^2^	*p*	r	*p*
		-	+					
CDX2	-	36	31	67	4.603	0.032*	0.334	0.001**
		(53.7%)	(46.3%)					
	+	11	24	35				
		(31.4%)	(68.6%)					

### CDX2 silencing downregulated Reg IV expression in gastric cancer cells

**Fig. 2:**
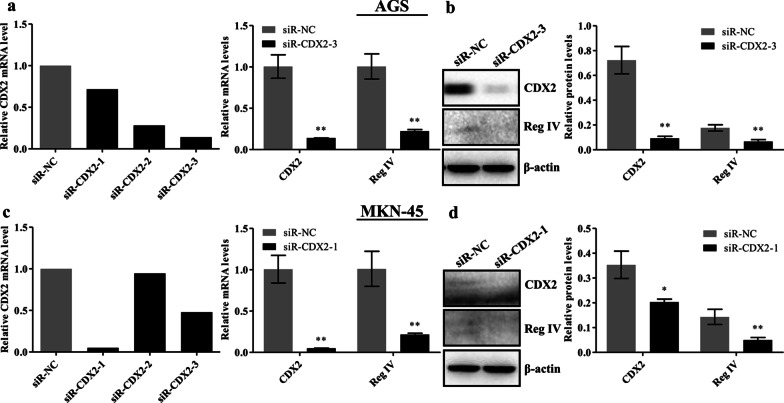
CDX2 silencing downregulated Reg IV in gastric cancer cells. AGS (**a**, **b**) and MKN-45 (**c**, **d**) cells were transfected with the three CDX2 siRNAs for 48 h, and the CDX2 siRNA with the optimal silencing effect was chosen and used for subsequent experiments (left of **a**, **c**). CDX2 mRNA level was measured using real-time PCR, and the results were normalized to GAPDH (right of **a**, **c**). Moreover, western blotting was used to analyze Reg IV and CDX2 protein levels, β-actin was used as a loading control. Representative immunoblots are shown and the relative protein signal intensity was quantitatively analyzed using Image Lab software (**b**, **d**). Full-length original blots are presented in Additional file 1. Data are presented as mean ± SD. *p < 0.05; **p < 0.01 was compared with the control groups

To confirm whether CDX2 regulates Reg IV expression in gastric cancer cells, CDX2 expression was knocked down via RNA interference (RNAi). Measurement of the relative CDX2 mRNA expression level at 48 h post-transfection with the three CDX2 siRNAs showed that siR-CDX2-3 produced an optimal interference effect and CDX2 mRNA levels decreased by 86.0% in AGS cells (Fig. 2a). siR-CDX2-3 significantly reduced the mRNA (*p* = 0.0002) and protein (*p* = 0.003) levels of Reg IV in AGS cells (Fig. 2a–b). In MKN-45 cells, siR-CDX2-1 achieved the highest knockdown effect. Reg IV mRNA (*p* = 0.001) and protein (*p* = 0.007) levels were also decreased in the CDX2 knockdown group compared with the control group (Fig. 2c–d). These results indicate that the silencing of CDX2 led to the downregulation of Reg IV in gastric cancer cells.

### CDX2 overexpression upregulated Reg IV expression in gastric cancer cells

**Fig. 3:**
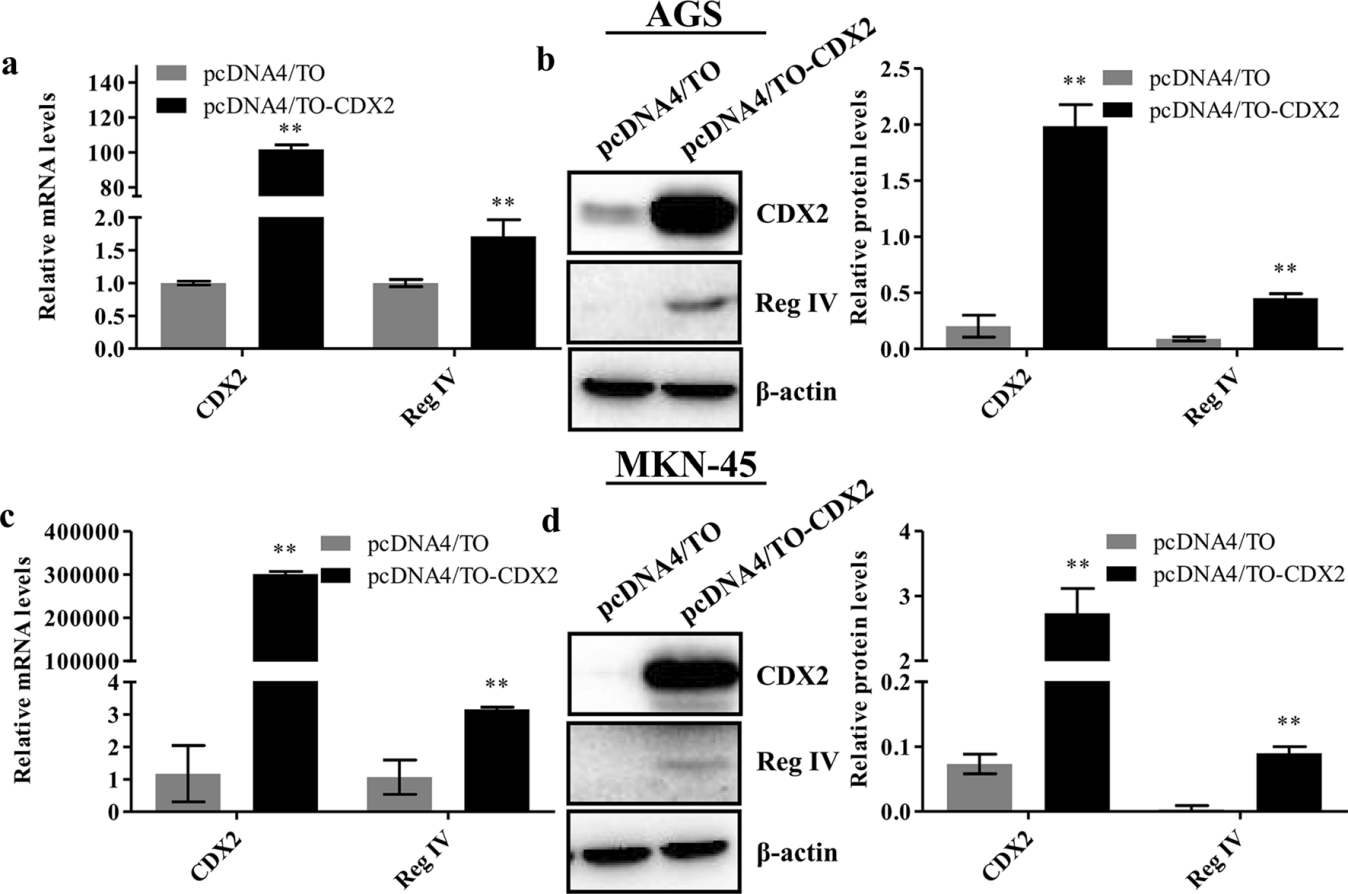
CDX2 overexpression upregulated Reg IV in gastric cancer cells. AGS (**a**, **b**) and MKN-45 (**c**, **d**) cells were transfected with pcDNA4/TO-CDX2 or pcDNA4/TO for 36 h. Reg IV and CDX2 mRNA levels were evaluated using real-time PCR, and the results were normalized to GAPDH (**a**, **c**). Reg IV and CDX2 protein expression were analyzed via western blotting. β-actin was used as a loading control. Representative immunoblots are shown, and the relative protein signal intensity was quantitatively analyzed using Image Lab software (**b**, **d**). Full-length original blots are presented in Additional file 1. Data are presented as mean ± SD. *p < 0.05; **p < 0.01 compared with the control groups

To further confirm the regulation of Reg IV by CDX2 in gastric cancer cells, cells were transfected with pcDNA4/TO-CDX2 or pcDNA4/TO (control) for 36 h and CDX2 expression was analyzed via real-time PCR and western blotting. Figure 3a, c shows that CDX2 mRNA levels increased by 101.8-fold (AGS, *p* < 0.0001) and 301,153.3-fold (MKN-45, *p* < 0.0001). The western blotting results further verified the overexpression of CDX2 protein in AGS (*p* = 0.0001) and MKN-45 (*p* = 0.007) cells (Fig. 3b, d). We found that Reg IV mRNA and protein levels increased after CDX2 overexpression in AGS and MKN-45 cells (*p* < 0.01; Fig. 3a–d). These results suggest that the overexpression of CDX2 led to the upregulation of Reg IV in gastric cancer cells.

### Reg IV did not affect CDX2 expression in gastric cancer cells

**Fig. 4:**
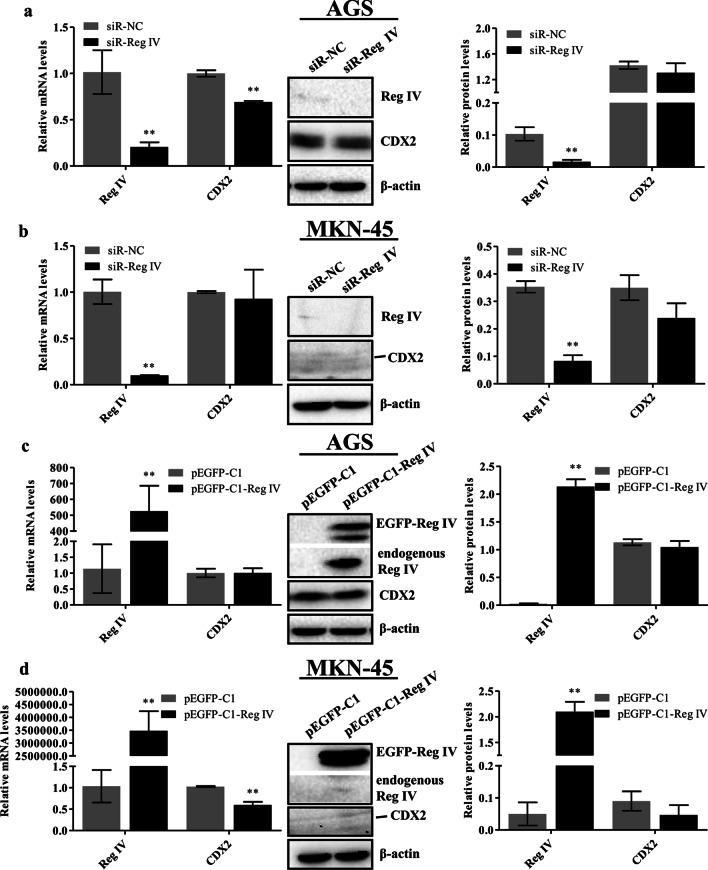
Reg IV had no effect on CDX2 in gastric cancer cells. AGS and MKN-45 cells were transfected with siR-Reg IV (**a**, **b**) or pEGFP-C1-Reg IV (**c**, **d**). Reg IV and CDX2 mRNA levels were evaluated using real-time PCR, and relative mRNA expression results were normalized to GAPDH (left of **a**–**d**). Reg IV and CDX2 protein expression levels were analyzed via western blotting. β-actin was used as a loading control. Representative immunoblots are shown in the middle panels of **a**–**d**. The relative protein signal intensity was quantitatively analyzed using Image Lab software and shown as histograms (right of **a**–**d**). Full-length original blots are presented in Additional file 1. Data are presented as mean ± SD. *p < 0.05; **p < 0.01 compared with the control groups

To investigate the effect of Reg IV on CDX2 in gastric cancer cells, Reg IV was silenced or overexpressed. siR-Reg IV, which had a significant silencing effect in our previous study [1], was transiently transfected into cells for 48 h. Real-time PCR analysis showed that Reg IV mRNA levels decreased by 79.5% (AGS) and 90% (MKN-45) (*p* < 0.01; left panels of Fig. 4a, b). The western blotting results also demonstrated that siR-Reg IV significantly reduced Reg IV expression compared with the control groups (*p* < 0.01; middle and right panels of Fig. 4a, b). Although the CDX2 mRNA level was downregulated (*p* < 0.0001), the protein level did not change (*p* = 0.274) following Reg IV silencing in AGS cells (Fig. 4a). However, Reg IV silencing had no effect on CDX2 mRNA and protein levels in MKN-45 cells (*p* > 0.05; Fig. 4b). These results suggest that Reg IV silencing had no effect on CDX2 expression in gastric cancer cells.

Cells were also transfected with pEGFP-C1-Reg IV or pEGFP-C1 (control), and the expression of Reg IV was validated by real-time PCR and western blotting. Figure 4c shows that Reg IV mRNA levels increased by 526.1-fold (AGS) and 3,478,357.1-fold (MKN-45) (*p* < 0.01). To further determine whether the Reg IV protein was overexpressed, we detected the fusion protein (EGFP-Reg IV; ~ 41 KDa) of EGFP (27 KDa) and Reg IV (14 KDa) via western blotting using the Reg IV antibody. The results showed that EGFP-Reg IV was expressed when AGS and MKN-45 cells were transfected with pEGFP-C1-Reg IV (Fig. 4c, d). Moreover, the endogenous Reg IV level was significantly upregulated in pEGFP-C1-Reg IV-transfected AGS cells (Fig. 4c). However, CDX2 mRNA and protein levels were unchanged following Reg IV overexpression in AGS cells (*p* > 0.05; Fig. 4c). Although Reg IV overexpression downregulated CDX2 mRNA levels in MKN-45 cells (*p* = 0.0002), protein expression did not change (*p* = 0.154; Fig. 4d). These results indicate that Reg IV overexpression had no effect on CDX2 expression in gastric cancer cells.

### CDX2 promoted the migration and invasion of gastric cancer cells

**Fig. 5:**
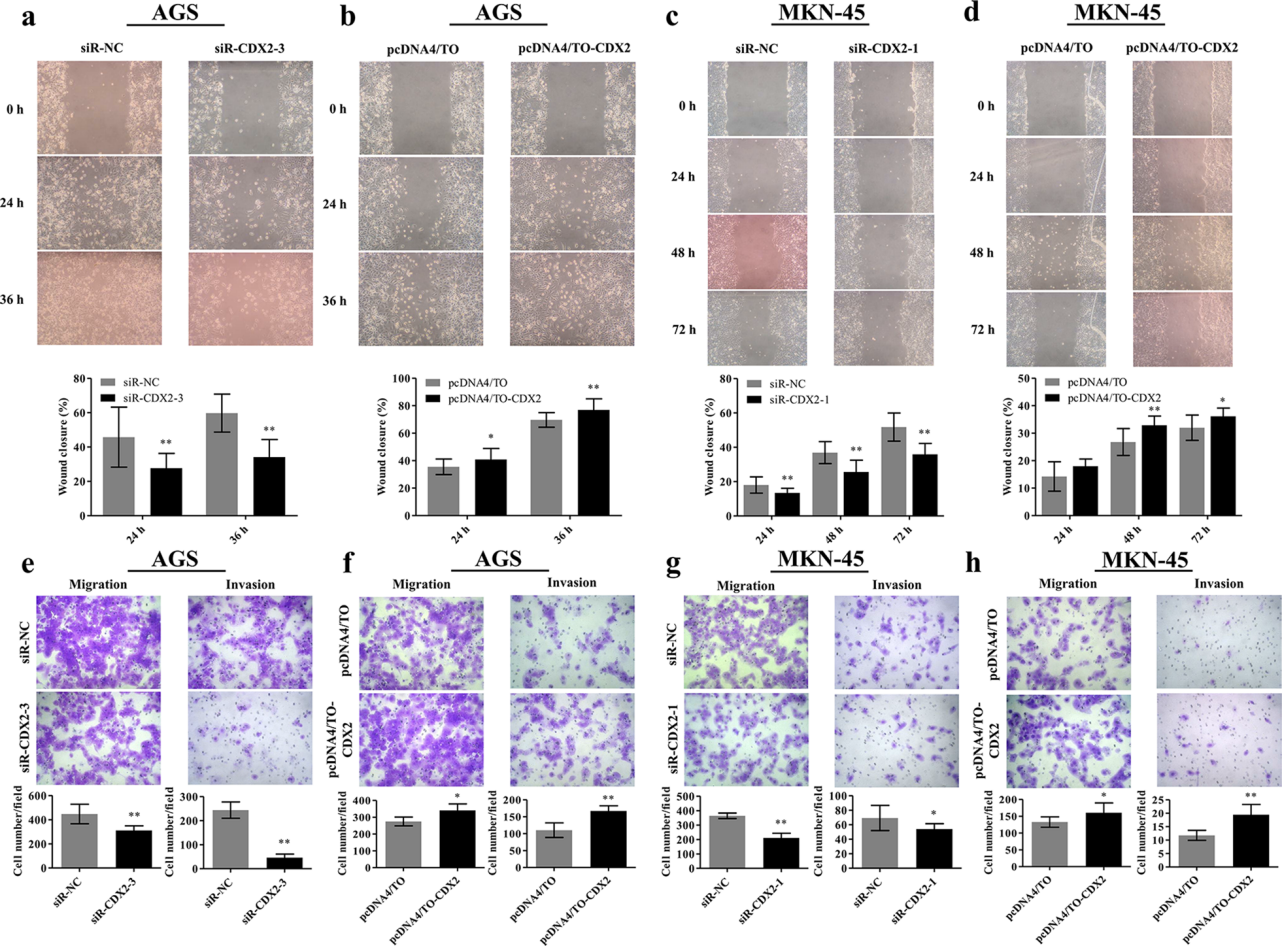
CDX2 promoted the migration and invasion of gastric cancer cells. **a**–**d** Wound-healing assay to detect the migration of AGS and MKN-45 cells after CDX2 silencing or overexpression. Representative microscopic images are shown. Magnification 100 × . The bar charts show the percentage of wound-healing at indicated times (below panel). **e**–**h** Effects of CDX2 silencing or overexpression on the migration and invasion of both cell lines were assessed by Transwell assay. Cells transfected with CDX2 siRNA or pcDNA4/TO-CDX2 migrated across uncoated or Matrigel-coated Transwell inserts. Cells in five fields from each membrane were counted using an optical microscope. Representative images of migrated cells are shown. Magnification 200×. The bar charts show the number of cells that migrated across 8 µm pores to the underside of the membrane. Data are presented as mean ± SD. *p < 0.05; **p < 0.01 compared with the control groups

To determine the roles of CDX2 in the motility of AGS and MKN-45 cells, wound-healing, Transwell migration, and Matrigel invasion assays were performed following CDX2 silencing or overexpression. In the wound-healing assay, migration of AGS cells was significantly reduced after CDX2 silencing in comparison with the control group (*p* = 0.002 for 24 h, *p* < 0.0001 for 36 h; Fig. 5a). Alternatively, cell migration was significantly increased after CDX2 overexpression (*p* = 0.047 for 24 h, *p* = 0.008 for 36 h; Fig. 5b). Because MKN-45 cells migrated very slowly, the wound healing process was monitored for 72 h. Consistent with the findings in AGS cells, knockdown of CDX2 dramatically inhibited the migration of AGS cells (*p* < 0.01; Fig. 5c) while overexpression of CDX2 significantly increased migration, especially after 48 h (*p* = 0.003) and 72 h (*p* = 0.031) (Fig. 5d). Moreover, the Transwell migration assay showed that CDX2 silencing significantly reduced the number of AGS cells that migrated from the upper surface to the lower surface of the inserts (*p* = 0.009; Fig. 5e), and CDX2 overexpression had the opposite effect (*p* = 0.014; Fig. 5f). Similar results were also observed in MKN-45 cells (Fig. 5g–h). Therefore, these data indicate that CDX2 promoted the migration of gastric cancer cells.

We further assessed the impact of CDX2 on the invasive properties of AGS and MKN-45 cells using Matrigel-coated inserts. Compared with the control group, the number of cells that passed through the Matrigel-coated membranes reduced by 81.0% in AGS cells transfected with siR-CDX2-3 (*p* < 0.0001; Fig. 5e). However, CDX2 overexpression resulted in the opposite effect (*p* = 0.0002; Fig. 5f). Consistently, CDX2 silencing inhibited the invasion of MKN-45 cells (*p* = 0.015; Fig. 5g), and CDX2 overexpression markedly increased invasion (*p* < 0.0001; Fig. 5h). These results show that CDX2 promoted the invasion of gastric cancer cells.

## Discussion

Gastric cancer is the fifth most commonly diagnosed cancer and the third leading cause of cancer deaths in men and women worldwide [43]. It is a complex, heterogeneous, and multifactorial disease with different phenotypes [44]. Most gastric cancers are adenocarcinomas that can be histologically divided into intestinal, diffuse, mixed, and indeterminate subtypes and vary in morphology, epidemiology, progression pattern, genetics, and clinical type. The most common subtype is intestinal, which occurs in approximately 54% of cases. Under normal conditions, CDX2 expression is restricted to the intestine. However, it is ectopically expressed in IM lesions of the stomach and intestinal-type gastric carcinoma [1–8]. Several studies have reported that CDX2 acts as a tumor suppressor in gastric cancer [6–8, 21–31]. However, others consider CDX2 an oncogene in gastric cancer [1, 2, 9–20]. These contradictory findings imply that CDX2 plays a complex regulatory role in gastric cancer. It was reported that Reg IV promoted tumor cell proliferation, metastasis, and migration and inhibited apoptosis in gastric cancer [36–39]. Collectively, although CDX2 and Reg IV are involved in the development and progression of gastric cancer, the regulatory correlation between CDX2 and Reg IV in gastric cancer remains unclear.

Results from this study demonstrated the following: First, there was a significantly positive correlation between the expression of CDX2 and Reg IV at the mRNA and protein levels in gastric cancer tissues. Previous studies have shown that CDX2 expression is associated with Reg IV expression [41, 45]. Yasui et al. reported that all of the Reg IV positive cells were also positive for CDX2 in both IM and gastric cancer; however, CDX2 positive cells were not consistently positive for Reg IV. This indicates that CDX2 might be an upstream regulator of Reg IV expression [41]. Another recent study showed that Reg IV expression was observed in all CDX2 positive cases of ovarian mucinous cancer [45]. Our study further demonstrated that CDX2 expression was positively correlated with Reg IV expression in gastric cancer tissues. Second, CDX2 upregulated Reg IV expression in gastric cancer cells. Previous studies demonstrated that Reg IV expression was induced by glioma-associated oncogene homolog 1 (GLI1) in pancreatic cancer [46] or by growth factors including transforming growth factor-α (TGF-α), epidermal growth factor (EGF), basic fibroblast growth factor (bFGF), and hepatocyte growth factor (HGF) in colon cancer [47]. In the present study, Reg IV expression was also positively regulated by CDX2 in gastric cancer cells. Consistent with our findings, previous studies reported that Reg IV expression was induced by CDX2 overexpression in OMC-3 ovarian mucinous carcinoma cells and HT-29 colon cancer cells [42, 45] and suppressed by CDX2 siRNAs in HSC-39 gastric cancer cells [42]. Third, Reg IV had no effect on CDX2 expression in gastric cancer cells. SOX9 is a transcription factor which belongs to the SOX family and was induced by Reg IV in MKN-28, MKN-45, and AGS gastric cancer cells [40, 41]. CDX2 is also a transcription factor; however, silencing and overexpression of Reg IV did not affect CDX2 expression in AGS and MKN-45 cells. Fourth, CDX2 promoted migration and invasion of gastric cancer cells. The same research group has shown that CDX2 siRNA significantly inhibited cell proliferation, caused G0/G1 phase cell cycle arrest, induced apoptosis, and decreased migration and invasion in MGC-803 cells [19], whereas CDX2 overexpression produced results similar to those of CDX2 siRNA in MGC-803 cells [28], suggesting that CDX2 may play a dual role in the regulation of cell growth and death in MGC-803 cells. However, another study showed that CDX2 overexpression suppressed cell migration and invasion in MGC-803 cells and that CDX2 silencing promoted migration and invasion in NCI-N87 cells [26], indicating that CDX2 may inhibit migration and invasion of gastric cancer cells. Moreover, another report showed that ectopic expression of CDX2 reduced migration and invasion in MKN-45 cells [29], which is contrary to our findings in MKN-45 cells. The inconsistency in these results may be due to the different gastric cancer cell lines used in the studies. We previously found that Reg IV overexpression upregulated SOX9 and promoted migration and invasiveness of tumor cells, while Reg IV silencing produced opposite results. In addition, SOX9 silencing inhibited the migration and invasion in MKN-45 and AGS cells, demonstrating that Reg IV may promote invasion and migration of MKN-45 and AGS cells through upregulation of SOX9 in AGS and MKN-45 cells [40]. In this study, CDX2 overexpression upregulated Reg IV expression and enhanced migration and invasion and CDX2 silencing downregulated Reg IV expression and suppressed migration and invasion in AGS and MKN-45 cells. These results demonstrate that CDX2 may promote cell migration and invasion through upregulation of Reg IV/SOX9 signaling in AGS and MKN-45 cells. Nevertheless, our and other studies also showed that silencing of SOX9 upregulated Reg IV protein expression in AGS, MKN-45, and MKN-74 cells [40, 41]. Furthermore, a previous report concluded that SOX9 also inhibited CDX2 protein expression both in intestinal adenocarcinoma cells in vitro and in a nude mouse xenograft tumor model [48]. Thus, we speculate that the aberrant expression of SOX9 may trigger negative feedback regulation on CDX2 and Reg IV in gastric cancer cells. Specifically, the ectopic expression of CDX2 may increase Reg IV and SOX9 levels, leading to the enhancement of migration and invasion. When SOX9 is increased to a certain level, it may conversely suppress the expression of CDX2 and Reg IV, resulting in a reduction of migration and invasion of gastric cancer cells. Further studies are needed to confirm these hypotheses.

## Conclusions

Our study demonstrated that CDX2 expression was positively correlated with that of Reg IV in gastric cancer. Moreover, CDX2 promoted the migration and invasion of gastric cancer cells (AGS and MKN-45) through upregulation of Reg IV expression. Consequently, CDX2 may have considerable potential as a novel therapeutic target for gastric cancer.

## Supplementary Information

**Additional file 1: Figure S1.** Original blots of CDX2 for Figs. 2–4. **(a)** Fig. 2b and Fig. 3b. **(b)** Fig. 4a. **(c)** Fig. 4c. (**d)** Fig. 3d. **(e)** Fig. 2d, Fig. 4b, and Fig. 4d. Any lanes not included in the final figures or not related to the results in this manuscript were not marked on the original blot images. **Figure S2.** Original blots of Reg IV for Figs. 2–4. **(a)** Fig. 4a and Fig. 4c. **(b)** Fig. 3b. **(c)** Fig. 2b. (**d–g**) Fig. 3d and Fig. 4d. Exposure times were 2 s, 14.1 s, 38.3 s, and 607.0 s, respectively. **(h)** Fig. 4b. **(i)** Fig. 2d. Any lanes not included in the final figures or not related to the results in this manuscript were not marked on the original blot images. **Figure S3.** Original blots of β-actin for Figs. 2–4. **(a)** Fig. 2b and Fig. 3b. **(b)** Fig. 4a and Fig. 4c. **(c)** Fig. 3d and Fig. 4d. **(d)** Fig. 2d. Any lanes not included in the final figures or not related to the results in this manuscript were not marked on the original blot images.

## Data Availability

All data generated or analysed during this study are included in this published article.

## Abbreviations

IHC: Immunohistochemistry
CDX2: Caudal type homeobox 2
IM: Intestinal metaplasia
MDR: Multidrug resistance
REG4: Regenerating islet-derived family member 4
GPR37: G protein-coupled receptor 37
SOX9: SRY related high-mobility group box 9
Ct: Cycle threshold
FBS: Fetal bovine serum
GLI1: Glioma-associated oncogene homolog 1
TGF-α: Transforming growth factor-α
EGF: Epidermal growth factor
bFGF: Basic fibroblast growth factor
HGF: Hepatocyte growth factor
