# Supplementary material for: CDX2 and Reg IV expression and correlation in gastric cancer
Source: BMC Gastroenterol. 2021 Feb 27;21:92. doi: 10.1186/s12876-021-01678-9 (PMC7913228; doi:10.1186/s12876-021-01678-9)
Supplement: Supplementary file 1 — Additional file 1: Figure S1. Original blots of CDX2 for Figs. 2–4. (a) Fig. 2b and Fig. 3b. (b) Fig. 4a. (c) Fig. 4c. (d) Fig. 3d. (e) Fig. 2d, Fig. 4b, and Fig. 4d. Any lanes not included in the final figures or not related to the results in this manuscript were not marked on the original blot images. Figure S2. Original blots of Reg IV for Figs. 2–4. (a) Fig. 4a and Fig. 4c. (b) Fig. 3b. (c) Fig. 2b. (d–g) Fig. 3d and Fig. 4d. Exposure times were 2 s, 14.1 s, 38.3 s, and 607.0 s, respectively. (h) Fig. 4b. (i) Fig. 2d. Any lanes not included in the final figures or not related to the results in this manuscript were not marked on the original blot images. Figure S3. Original blots of β-actin for Figs. 2–4. (a) Fig. 2b and Fig. 3b. (b) Fig. 4a and Fig. 4c. (c) Fig. 3d and Fig. 4d. (d) Fig. 2d. Any lanes not included in the final figures or not related to the results in this manuscript were not marked on the original blot images. [file 12876_2021_1678_MOESM1_ESM.docx]

**CDX2 and Reg IV expression and correlation in gastric cancer**

Dandan Chai, Huifen Du, Kesheng Li^*^, Xueliang Zhang, Xiaoqin Li, Xiaoning Zhao, Xiaowen Lian and Yang Xu


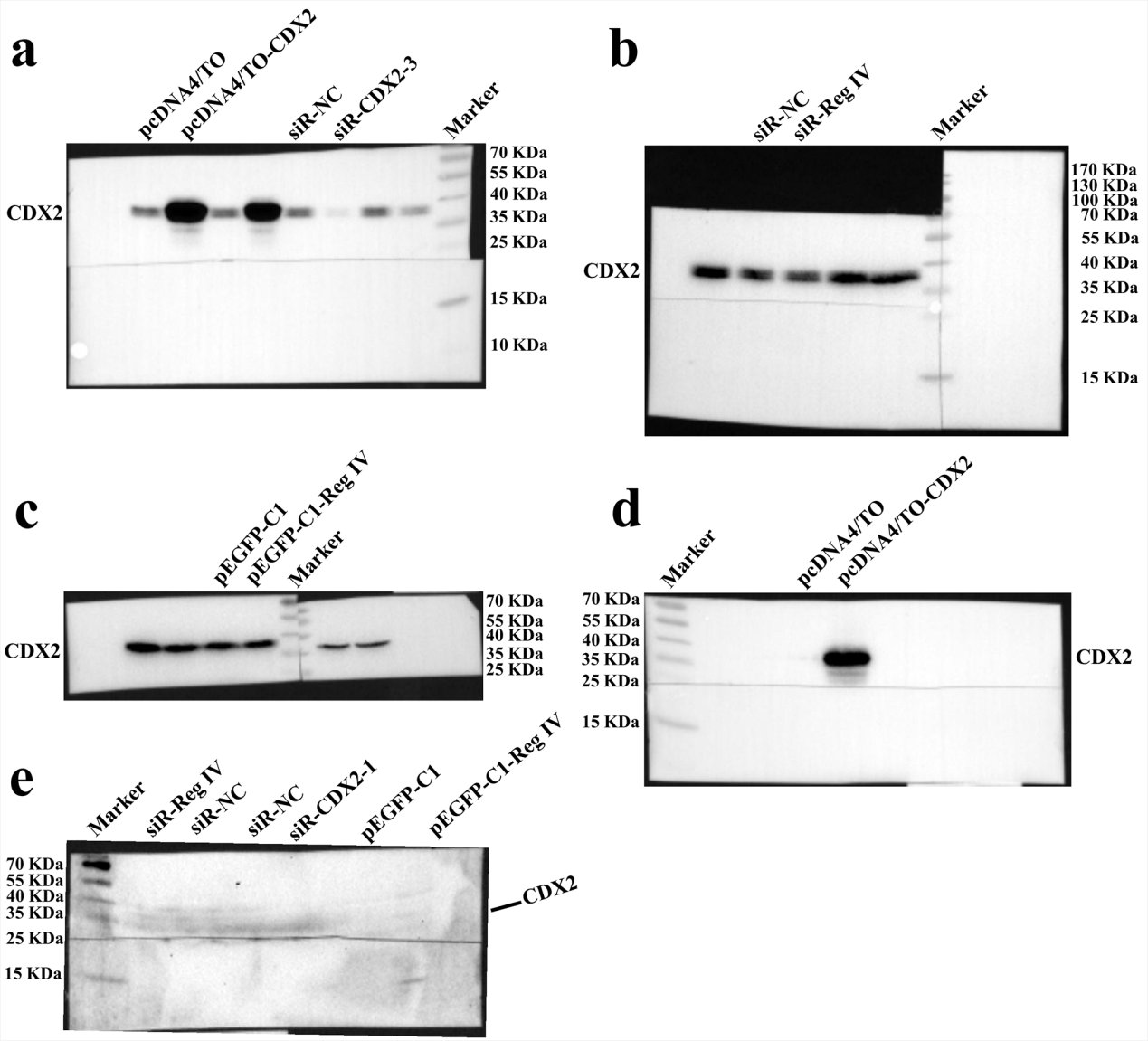


**Additional file 1: Figure S1.** Original blots of CDX2 for Fig. 2–4. (**a)** Fig. 2b and Fig. 3b. **(b)** Fig. 4a. **(c)** Fig. 4c. (**d)** Fig. 3d. **(e)** Fig. 2d, Fig. 4b, and Fig. 4d. Any lanes not included in the final figures or not related to the results in this manuscript were not marked on the original blot images.


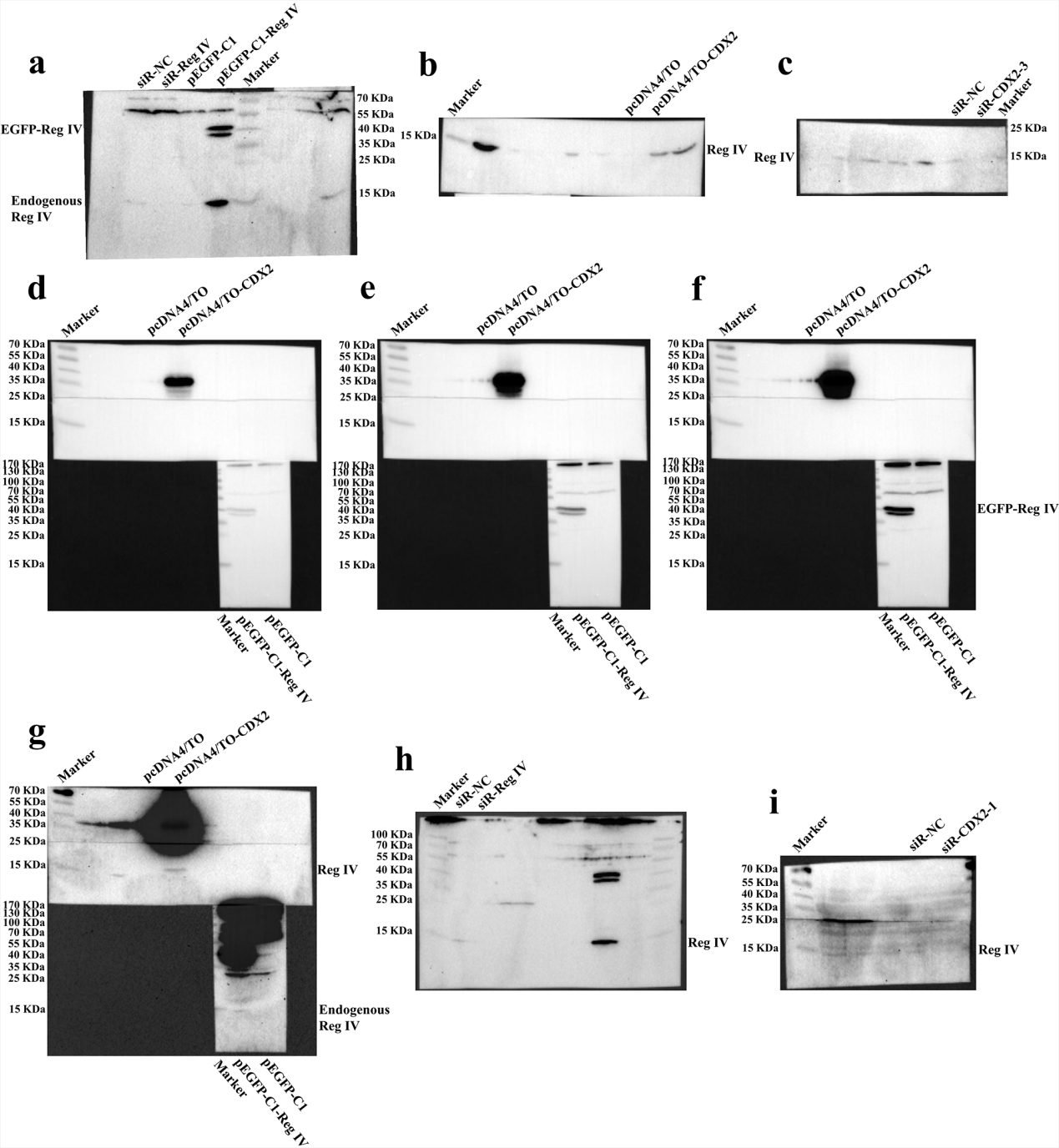


**Additional file 1: Figure S2** Original blots of Reg IV for Fig. 2–4. (**a**) Fig. 4a and Fig. 4c. **(b)** Fig. 3b. **(c)** Fig. 2b. (**d–g**) Fig. 3d and Fig. 4d. Exposure times were 2 s, 14.1 s, 38.3 s, and 607.0 s, respectively. **(h)** Fig. 4b. **(i)** Fig. 2d. Any lanes not included in the final figures or not related to the results in this manuscript were not marked on the original blot images.


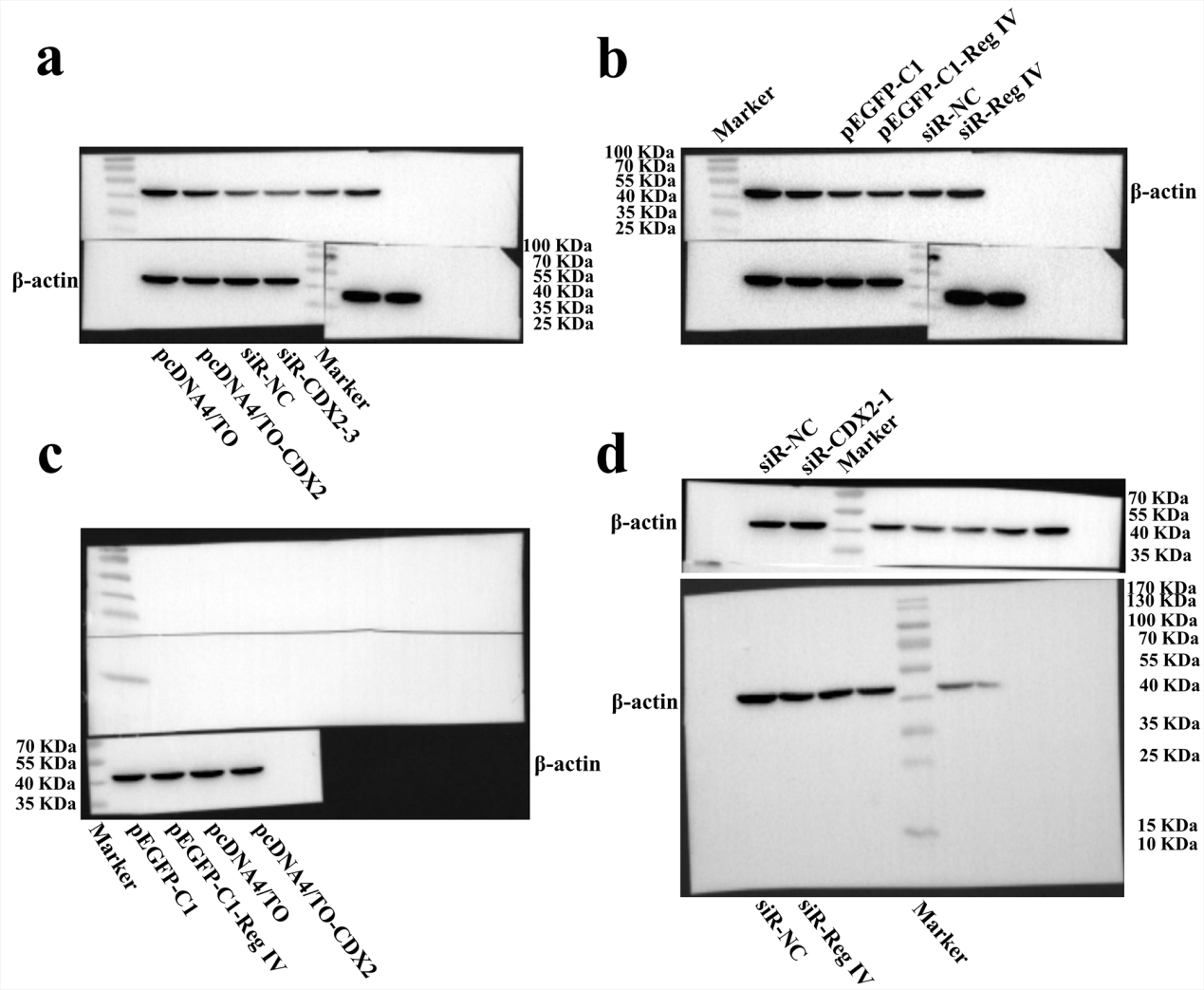


**Additional file 1: Figure S3** Original blots of β-actin for Fig. 2–4. **(a)** Fig. 2b and Fig. 3b. (**b**) Fig. 4a and Fig. 4c. **(c)** Fig. 3d and Fig. 4d. **(d)** Fig. 2d. Any lanes not included in the final figures or not related to the results in this manuscript were not marked on the original blot images.
